# Glances at the Hospitals

**Published:** 1904-01-16

**Authors:** 


### GLANCES AT THE HOSPITALS.

### ROYAL INFIRMARY, STIRLING.

THE income was £1,386 and the expenditure £1,300, leaving a balance of £86 in the treasurer's hands as the result of the year's working. The capital account exceeded £12,000, showing an increase of £1,404 on the previous year. Verily the governors are to be congratulated on the state of their finances. It is proposed to build a convalescent hospital which will prove a very useful adjunct to the Infirmary. We regret to notice that the workpeople's own subscriptions show a slight falling-off. This item of hospital income ought to be an ever-increasing one, and surely the working-men of Stirling and its neighbourhood ought to raise more than £131. At the beginning of the year there were 23 patients in the Infirmary, and 275 were admitted during it. Of the total 148 were discharged recovered, 94 were relieved, 13 not relieved, 26 died and 17 remained in the wards. The daily average number resident was 22, and the mortality was 9 2 per cent., but 14 of the fatal cases died within 48 hours of admission. The omission of these would greatly reduce the percentage. There is a complete *résumé* of all the cases under treatment, showing the age, sex, residence, occupation, disease, time in the hospital, and the results of treatment. In addition there is a tabulated statement of medical and surgical cases. There were 10 cases of acute pneumonia of which 6 recovered and 4 died. Of 10 cases of gastric ulcer, 8 recovered and 2 were under treatment at the date of the report. Of 3 cases of epilepsy all are put down as recovered. Concerning these we should like to have seen a note in the "Remarks" column stating the treatment and the length of time under observation after the recovery. There is no anæsthetic table.

### THE HASTINGS HOSPITAL.

THE ordinary income amounted to £3,794, being a decrease of £113 on the income of the previous year, and the expenditure amounted to £5,481, being an increase of £799. In addition to this there was an extraordinary expenditure of £587. The governors point out that the annual subscriptions and the interest from invested capital produce less than half of the expenditure, and they justly say that unless the income can be increased the hospital must be curtailed in some of its departments. They appeal most earnestly to the town and neighbourhood to assist them in providing for the sick poor, and it is to be desired that their appeal should be suitably answered by all classes. It would be interesting in this connection to learn what has been done to induce the workpeople to increase their own subscriptions, for assuredly nothing is so likely to stimulate the donations of the richer people than to learn that the people for whose benefit the institution exists are doing their best to help themselves. It does not appear whether there is any wage limit above which gratuitous advice is not given. This point might be considered. It appears that 4,883 patients were treated at the hospital during the year, being 225 over the number of the previous year. There were 62 patients in residence on January 1st, and 710 were admitted during it, making a total of 772 cases under treatment. Of these 535 were discharged cured or convalescent, 30 relieved, 58 for other reasons, 35 died, and 52 remained in the wards. The medical and surgical tables merely show the numbers treated for the various diseases, and hence are practically useless. The total number of operations was 372. There is no table showing the nature of the anæsthetics used. We also failed to find any statement of the average cost per bed occupied, or of the cost per head per week. The percentage of cases cured was 74·3, and the percentage mortality was 4·78.

### THE LUTON HOSPITAL.

THE income amounted to £1,473 and the expenditure to £1,362, showing a balance on the right side of about £110. It is intended to enlarge the hospital as a Coronation memorial. For this purpose a sum of £3,000 is required, and £2,540 already subscribed, so that the extensions will be at once proceeded with. The total number of in-patients treated during the year was 242. Of these 195 were discharged either cured or relieved, 6 not improved, 6 were discharged for other reasons, 22 died, and 13 remained in the wards. A complete list of the patients is given showing all essential particulars, and admirable as this plan is for tracing any individual case, it is not so useful as properly arranged tables would be for purposes of comparison. There is no anæsthetic table, nor did we notice any reference to the cost per bed occupied or to the cost per head per week.

### THE OXYGEN HOSPITAL, FITZROY SQUARE, W.

THE ordinary income was £1,438, and the ordinary expenditure £1,214. In all, 96 cases have been treated during the year, and of these 70 have been discharged, and 26 remained under treatment. We are told that the general work of the hospital in healing ulcers, wounds, burns, etc., has proved as successful as ever. During the six years or so of the hospital's existence it has treated 44 cases of lupus. Of these 22 cases have been discharged healed, and, as far as can be ascertained, have remained well for periods varying from one year to six years. Nine cases have been discharged healed, but the disease has recurred in periods of from six months to five years. Nine other cases have been discharged much improved, and in four the treatment failed to give any relief. The report states that these results compare favourably with any other form of treatment. During the year seven cases of middle-ear deafness were treated, and in all instances the hearing was greatly improved. A cubicle for the treatment of consumption has been erected. Here the air respired is drawn in by an exhaust fan, being dried, filtered, ozonised, and rarified before reaching the patient, who spends a large portion of the 24 hours in the cubicle. Five cases were subjected to this treatment, with the result that the disease was arrested in all; but, as the committee remark, the cure of consumption being problematical, the cases are tabulated in the "greatly relieved" column.

### CHESTER GENERAL INFIRMARY.

THE total ordinary income amounted to £4,917, and the total ordinary expenditure to £5,588, showing an adverse balance of about £670. The special receipts were £736, and the special expenditure £1,125, so that when the year closed there was a balance of nearly £1,300 due to the treasurer. On the first of January the hospital contained 83 patients, and 1,013 were admitted during the year, making a total of 1,096 under treatment. Of this number 705 were discharged cured, 203 relieved, 13 for other reasons, 89 died, and 86 remained in the wards. The mortality of the cases treated to a termination was 3·5 per cent. The medical and surgical tables are carefully compiled. We notice that of 35 cases of pneumonia 24 were cured, 1 was relieved, 6 died, and 4 were under treatment at the date of the report. Of 21 cases of appendicitis 18 were cured, and 3 remained under treatment. The operation for strangulated hernia was performed 21 times. Of these 12 were successful, 7 died, and 2 remained under treatment. There is no anæsthetic table.

### AYR COUNTY HOSPITAL.

THE ordinary income was £3,703, and the ordinary expenditure was £3,978. The extraordinary income was £738 and the extraordinary expenditure was £253. A legacy of £400 was placed to the capital account; and the deficit in the accounts amounted to £130, but this has been met out of a fund known as the Maintenance Reserve Fund. There were 68 patients in residence at the beginning of the year, and 774 were admitted during it, making a total of 842 under treatment. Of these 670 were cured or relieved, 49 not improved, 57 died, and 66 remained in the hospital. The average daily number of patients was 66; the average residence was 29 days. The cost of each bed occupied was £55 15s., and the average cost of each patient was £4 15s. 6d. The tables are very well arranged, and it is quite easy to pick out the various diseases and the results of treatment. There were 31 cases of pneumonia with 3 deaths; of 25 cases of diphtheria, 21 were cured and 4 died; of 122 cases of scarlet fever, 97 were cured, 6 died and 19 remained under treatment; of 36 cases of enteric fever, 32 recovered.

### THE SOUTHAMPTON HOSPITAL.

THE income was £5,600, and the expenditure was £8,054, which shows a very large deficit. The daily average number of patients was 101, the average stay in the hospital was $23\frac{1}{2}$ days, the cost per bed occupied was £71 3s., the cost per patient £4 11s. 9d., and the cost per patient per day was 3s. $9\frac{3}{4}$d. On the first of January the house contained 86 patients, and 1,480 were admitted during it, making a total of 1,566 under treatment. Of these 912 were discharged recovered, 286 relieved, 123 for various reasons, 108 died, and 111 remained under treatment. An analysis of the medical and surgical cases is given, and the operation table is carefully drawn out, but no anæsthetic table is printed.

### WEST CORNWALL MINERS' AND WOMEN'S HOSPITAL.

THE income amounted to £1,499, and the expenditure to £1,518, the latter representing the total cost of maintenance for the year. The total number of patients admitted was 338, and of this number 169 were men, 121 were women, and 48 were children. The average number resident was 30, and the average stay in the hospital was, for the men, 33 days, for the women 31 days, and for the children 35 days. The total cost per bed for the year was £49 8s., and the average cost for food per head per week was 6s.

### DURHAM COUNTY HOSPITAL.

THE income reached £3,942, and of this sum we are glad to be able to state that the workmen contributed £1,609. The expenditure was £2,975, so that the managers were able to transfer £500 to the deposit account, to repay the sum overdrawn at the beginning of the year, and to finish the year's work with a balance in hand of £299. Here is a state of finances all too rare in hospitals, and the Durham governors are to be congratulated on the excellence of their management. On the first of January the hospital contained 27 patients, and 534 were admitted during the year, making a total of 561 under treatment. Of these 387 were discharged cured, 89 relieved, 40 died, and 45 remained in the wards at the close of the year. The out-patients reached the respectable figure of 2,165, and 990 visits were paid to patients in their own homes. The medical and surgical tables are practically useless, as merely numbers without results are given. It is, for instance, merely tantalising to be told that 11 abdominal sections were performed, when we are left in ignorance of the number recovering. Nor is there any table of causes of death published, and we also miss a table of anæsthetics.

### LEEDS HOSPITAL FOR WOMEN AND CHILDREN.

DURING the year 387 in-patients were admitted. The average of each patient's stay in the hospital was 22 days, and the average number of beds occupied was 25. There were 11 deaths, giving a mortality of 3·20 per cent. The medical and surgical tables show the numbers "cured or relieved" and "died," and the operation table gives similar information. Ovariotomy was performed 5 times, all being successful. The total number of operations performed during the year was 364, and there were only 8 deaths. The anæsthetic table tells us that ether was used in 269 instances, chloroform in 76, a mixture of ether and chloroform in 14, sœmnoform in 3, and cocaine in 2.

